# The venom of South American rattlesnakes inhibits macrophage functions and is endowed with anti-inflammatory properties

**DOI:** 10.1155/S0962935196000038

**Published:** 1996-02

**Authors:** Maria C. C. de Sousa e Silva, Luis R. C. Gonçalves, Mario Mariano

**Affiliations:** 1 Laboratory of Pathophysiology Instituto Butantan Brazil; 2 Department of Immunology Institute of Biomedical Sciences Av. Prof. Lineu Prestes 2415 University of São Paulo São Paulo 05508-000 Brazil

## Abstract

The injection of *Crotalus durissus terrificus* venom into the foot pad of mice did not induce a significant inflammatory response as evaluated by oedema formation, increased vascular permeability and cell migration. The subcutaneous injection of the venom, or its addition to cell cultures, had an inhibitory effect on the spreading and phagocytosis of resident macrophages, without affecting the viability of the cells. This effect was not observed when the venom was added to cultures of thioglycollate elicited macrophages, but it was able to inhibit these macrophage functions when the cells were obtained from animals injected simultaneously with the venom and thioglycollate. These observations suggest that the venom interferes with the mechanisms of macrophage activation. Leukocyte migration induced by intraperitoneal injection of thioglycollate was also inhibited by previous venom injection. This down-regulatory activity of the venom on macrophage functions could account for the mild inflammatory response observed in the site of the snake bite in *Crotalus durissus terrificus* envenomation in man.


Mediators of Inflammation 5, 18–23 (1996)

### The venom of South American rattlesnakes inhibits macrophage functions and is endowed with anti-inflammatory properties

Maria C. C. de Sousa e Silva,[1]

Luis R. C. Gonçalves[1] and Mario Mariano[2,CA]

[1]Laboratory of Pathophysiology, Instituto Butantan; [2]Department of Immunology, Institute of Biomedical Sciences, Av. Prof. Lineu Prestes 2415, 05508-000, University of São Paulo, São Paulo, Brazil. Fax: (+55) 11 818 7224.

[CA]Corresponding Author

**THE injection of *Crotalus durissus terrificus* venom into the foot pad of mice did not induce a significant inflammatory response as evaluated by oedema formation, increased vascular permeability and cell migration. The subcutaneous injection of the venom, or its addition to cell cultures, had an inhibitory effect on the spreading and phagocytosis of resident macrophages, without affecting the viability of the cells. This effect was not observed when the venom was added to cultures of thioglycollate elicited macrophages, but it was able to inhibit these macrophage functions when the cells were obtained from animals injected simultaneously with the venom and thioglycollate. These observations suggest that the venom interferes with the mechanisms of macrophage activation. Leukocyte migration induced by intraperitoneal injection of thioglycollate was also inhibited by previous venom injection. This down-regulatory activity of the venom on macrophage functions could account for the mild inflammatory response observed in the site of the snake bite in *Crotalus durissus terrificus* envenomation in man.**

**Key words:** Anti-inflammatory, *Crotalus durissus terrificus*, Inflammation, Macrophages, Snake venom

### Introduction

For many years, clinical observations have demonstrated that the venom of Viperid snakes are highly phlogistic.[1,2] A classical exception, found among the genus *Crotalus*, is the South American rattlesnake, *Crotalus durissus terrificus* (Cdt). The venom of this snake does not induce significant inflammatory reaction at the site of the bite in human victims.[1,3] Moreover, recent studies have demonstrated that Cdt venom inhibits inflammatory signals like pain and oedema in some experimental models.[4,5]

Based on this information, the present study was undertaken in order to investigate some parameters of the inflammatory response experimentally induced in mice by Cdt venom injection, as well as the effect of this venom on some activities of resident peritoneal and inflammatory macrophages, an important component of the inflammatory process.

### Material and Methods

*Animals:* Male mice from the Swiss NIH strain and Nu/Nu mice from the Balb/c strain, weighing 18–22 g, were used throughout this study. The animals were kept with free access to food and water.

*Venom:* A pool of lyophilized venom was obtained from various adult specimens of *Crotalus durissus terrificus* snakes at the Laboratory of Herpetology of the Instituto Butantan. The venom solutions were prepared (w/v) in sterile saline, immediately before use.

*Histological analysis:* Histological sections of tissues of the abdominal wall of mice injected s.c. with 1.5 μg (50 μl) of Cdt venom were analysed by using light microscopy. The tissues were collected 1, 2, 4 and 6 h after venom injection, fixed in Bouin fixative and processed for embedding in paraffin. Histological sections were stained with Haematoxilin–eosin.

*Vascular permeability evaluation:* Vascular permeability was evaluated by extravasation of Evans blue in animals injected in the foot pad with 1.5 μg of Cdt venom. Animals were injected i.v. with a 2.5% solution of Evans blue (100 mg/kg of body weight), immediately after venom injection or at different times. Twenty min after the injection of the dye the animals were anaesthetized and sacrificed. The paw was cut off in the tibio–tarsic junction and the fragments were placed in tubes containing 2.0 ml of formamide (Merck) maintained for 48 h at 37°C for complete extraction of the dye.

© 1996 Rapid Science Publishers

The amount of extravased dye was estimated by using a spectrophotometer (Micronal B382, Brazil) to determine the absorbance at 618 nm, and comparing the result with a standard curve for the dye.

*Evaluation of paw oedema:* Animals were injected into the subplantar surface of the left hind paw with 50 µl of sterile saline containing different concentrations of Cdt venom. The contralateral paw received the same volume of sterile saline. The paw oedema was measured by plethysmography[6] at different times after the venom injection. The results were calculated as the difference between the volume of both paws and oedema was expressed as the percentage increase in paw volume. A group of mice was treated by the i.p. route with 10.0 mg/kg of promethazine (Sigma), 30 min before the injection of venom (1.5 µg).

*Cell migration:* The migration of cells into the peritoneal cavity of mice 2, 4, 6 and 24 h after the i.p. injection of 0.5 µg of venom was analysed by counting the total cells in a Neubauer's chamber and by the differential count in blood smears stained with panchromatic dye.[7]

*Peritoneal cells:* Animals were anaesthetized with ether and sacrificed by exsanguination by sectioning the cervical vessels. The peritoneal cavity was washed with 3 ml of cold phosphate-buffered saline (PBS), pH 7.4. After a gentle massage of the abdominal wall, the peritoneal fluid was collected in a plastic syringe. In some experiments 4% thioglycollate was injected into the peritoneal cavity and inflammatory peritoneal cells were harvested 4 days later. Cell viability was assessed by the Trypan blue exclusion test. Total and differential counts of peritoneal cell suspensions were made as above.

*Spreading of peritoneal macrophages:* The spreading capacity of peritoneal macrophages was estimated according to the method described by Rabinovitch and De Stefano.[8] Briefly, 50 µl of cell suspension ($1 \times 10^6$/ml) was placed onto glass coverslips and left to adhere for 10 min at room temperature. The coverslips were washed with PBS and incubated in medium 199 (with Earle's salts and L-glutamine) at 37°C for 1 h. Cells were fixed in a 2.5% glutaraldehyde solution and the index of spreaded cells was determined by examination under phase contrast microscopy. The spreading activity index was defined as the ratio between spreaded cells and 200 cells counted.

*Phagocytic activity of peritoneal macrophages:* A suspension of sheep erythrocytes, sensitized with rabbit antiserum against sheep erythrocytes, was placed over the coverslips with adhered peritoneal cells, for 30 min at 37°C, in an atmosphere containing 5% $CO_2$.[9] A total of 200 cells were counted, and the phagocytic activity index was calculated as the number of macrophages which had phagocytosed at least five erythrocytes.

*Effect of venom on macrophage functions* in vitro*:* Resident peritoneal macrophages were incubated with 0, 0.06, 0.125, 0.25, 0.5 and 1.0 µg of Cdt venom/ml of 199 culture medium. In one experiment, instead of Cdt venom, the culture medium was supplemented with 30% of serum obtained from mice injected s.c., 4 days before, with 1.5 µg of Cdt venom. The spreading and phagocytic activity indices of these cells were determined.

*Effect of venom on macrophage functions* in vivo*:* The spreading and phagocytic indices of peritoneal macrophages obtained from mice treated as follows, were determined: (a) previous i.p. injection of 0.5 µg Cdt venom (1, 2, 4, 8, 16, and 32 days); (b) previous s.c. injection of 1.5 µg Cdt venom (1, 2, 4, 8, 16, 32 days); (c) previous i.p. injection of 1.0 ml 4% thioglycollate concomitant with s.c. injection of 1.5 µg Cdt venom (4 days); (d) athymic nude mice, 4 days after s.c. injection of 1.5 µg Cdt venom; and (e) control groups injected with saline in the same volume and by the same routes.

*Effect of venom on the inflammatory response induced by thioglycollate:* The migration of cells into the peritoneal cavity 4 h after the injection of 4% thioglycollate (1.0 ml i.p.) was investigated in groups injected s.c. 1 h earlier with 1.5 µg Cdt venom, and compared to control groups injected with saline.

*Statistical analysis:* Data were expressed as the mean ± S.E.M. Student's *t*-test for impaired samples and *F*-test[10] were used to compare experimental and the respective control groups. Difference of results were considered statistically significant when $p < 0.05$.

### Results

*Inflammatory response induced by the venom:*

Peritoneal cavity. The cell migration to the peritoneal cavity induced by Cdt venom was similar to the control group in all times analysed (Table 1).

**Table 1.** Total and differential count of cells from the peritoneal cavity of mice injected with 0.5 μg *C.d.terrificus* (Cdt) venom (i.p.), compared to a control group injected with saline by the same route

| Time (h) | Group | Cell count ($\times 10^6$/ml) | | |
|---|---|---|---|---|
| | | Total | Differential | |
| | | | Mono | PMN |
| 2 | Saline | 2.32 ± 0.18 | 2.07 ± 0.18 | 0.18 ± 0.08 |
| | Cdt | 1.91 ± 0.15 | 1.81 ± 0.15 | 0.05 ± 0.01 |
| 4 | Saline | 2.65 ± 0.24 | 2.30 ± 0.80 | 0.34 ± 0.06 |
| | Cdt | 2.22 ± 0.07 | 2.00 ± 0.08 | 0.18 ± 0.05 |
| 6 | Saline | 1.64 ± 0.13 | 1.53 ± 0.12 | 0.08 ± 0.01 |
| | Cdt | 1.93 ± 0.24 | 1.82 ± 0.26 | 0.07 ± 0.01 |
| 24 | Saline | 1.75 ± 0.20 | 1.63 ± 0.22 | 0.08 ± 0.03 |
| | Cdt | 1.75 ± 0.17 | 1.68 ± 0.10 | 0.02 ± 0.01 |

Mono = mononuclear cells; PMN = polymorphonuclear cells.
Results expressed as mean ± S.E.M. $n = 4-7$ mice.

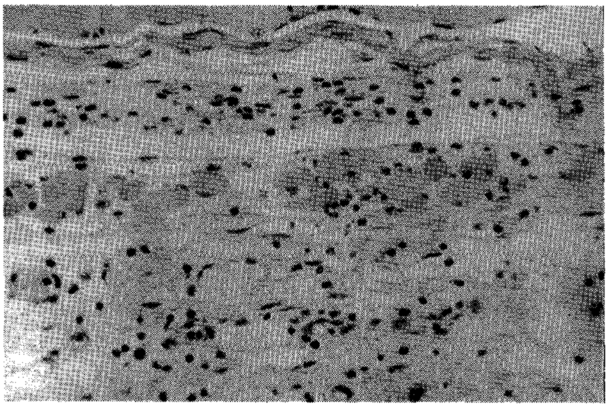

FIG. 1. Photomicrography of connective tissue of mice 6 h after the injection of 1.5 μg *C.d.terrificus* venom, showing a mild infiltrate of inflammatory cells. H.E. (450 ×).

Subcutaneous tissue. The histological analysis of the sites of inoculation of the Cdt venom showed a mild inflammatory response characterized by the presence of small cellular infiltrate and oedema. The cellular infiltrate was predominantly composed of polymorphonuclear cells and was a maximum 6 h after the venom injection (Fig. 1).

*Vascular permeability:* The s.c. injection of 1.5 μg of venom in the foot pad of mice caused a slight increase in vascular permeability that started immediately after the injection and decreased thereafter (Fig. 2).

*Paw oedema:* The oedematous response induced by intraplantar injection of Cdt venom (1.5 to 6.0 μg/paw) is shown in Fig. 3. The response was not dose-dependent. The peak of a mild response was observed 30 min after the venom injection. It was gradually reduced at subsequent times and completely disappeared 5 h later. Previous treatment of the animals with prometazin completely inhibited the mild oedematogenic effect of the venom (Fig. 3).

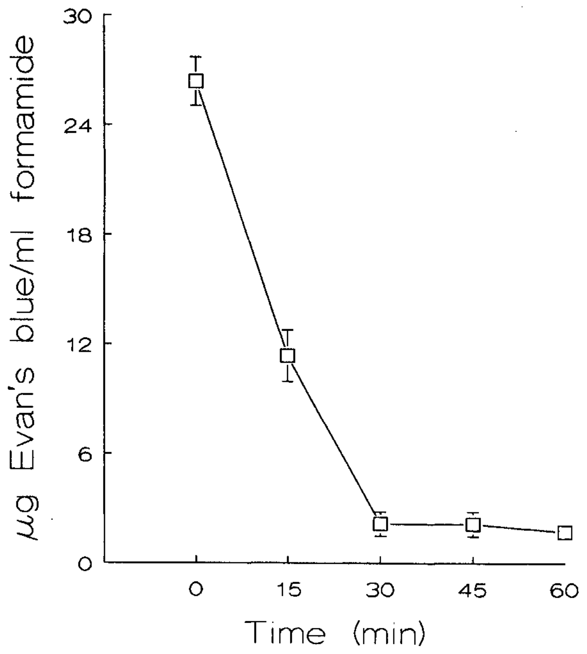

FIG. 2. Time-course of vascular permeability induced by the s.c. injection of 1.5 μg *C.d.terrificus* venom in paw of mice. Each point is the mean ± S.E.M. of four mice.

*Effect of Cdt venom on peritoneal macrophage spreading and phagocytosis* in vitro*:* As shown in Fig. 4, the addition of Cdt venom to culture medium at the concentration of 0.5 or 1.0 μg caused significant reduction in the spreading index of resident peritoneal macrophages when compared to a control group. The inhibitory effect on spreading and phagocytosis of the venom was not observed when it was added to cultures of inflammatory peritoneal macrophages elicited by thioglycollate (Fig. 4). The addition of serum from animals injected with Cdt venom to the culture medium did not alter the spreading index of the cells.

*Effect of* in vivo *administration of Cdt venom on peritoneal macrophage spreading and phagocytosis:* The i.p. or s.c. injection of the venom into normal mice induced a significant inhibition of peritoneal macrophage spreading after different time intervals (Fig. 5). A maximal inhibition was observed after 4 days of venom injection and to a lesser extent even after 16 days. The same inhibitory effect was observed on the phagocytic activity of these cells. The injection of Cdt venom in athymic nude mice also determined a significant inhibition of peritoneal macrophage spreading when compared to a control group.

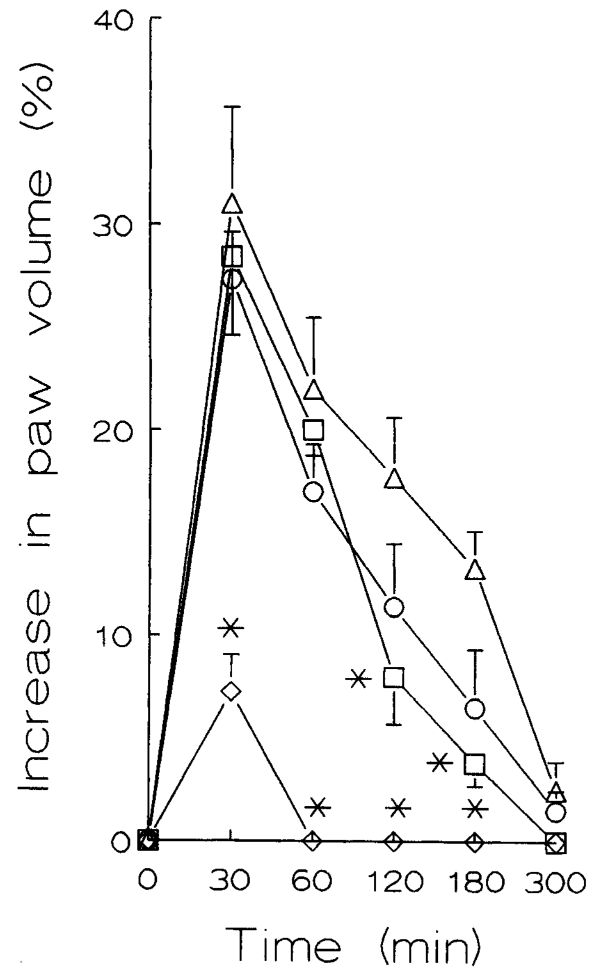

FIG. 3. Paw oedema induced by the injection of different concentrations of *C.d.terrificus* venom in untreated groups of mice. One group was treated with promethazine (Promet., 10 mg/kg, i.p., 30 min before) and injected with 1.5 µg Cdt venom. Each point is the mean ± S.E.M. of five to eight mice. *$p < 0.05$. Cdt venom: △, 1.5 µg; ○, 3.0 µg; □, 6.0 µg. Promethazine, ◇.

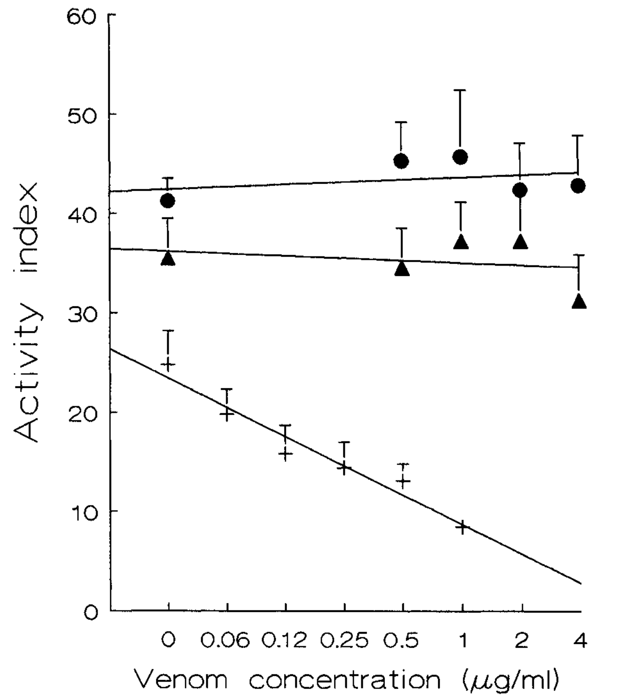

FIG. 4. Addition of different concentrations of *C.d.terrificus* venom to cultures of peritoneal macrophages. Effect on the spreading activity of resident (SP-re, +) and inflammatory (Sp-in, ▲) cells, and on phagocytosis of inflammatory (Pha-in, ●) cells. Each point is the mean ± S.E.M. of cultures from five mice. *$p < 0.05$.

*Effect of the venom on inflammatory cell migration:* The injection of Cdt venom (1.5 µg, s.c.) 1 h before the i.p. injection of thioglycollate, induced a significant reduction in the total number of cells in the peritoneal cavity 4 h after thioglycollate injection when compared to control animals not injected with the venom (Table 2). The differential count of these cells showed a significant reduction in the number of polymorphonuclear cells.

*Influence of venom administration on thioglycollate elicited activities of peritoneal cells:* A significant reduction of both phagocytic and spreading index of peritoneal macrophages was observed 4 days after the concomitant injection of venom (1.5 µg s.c.) and of thioglycollate solution (i.p.) in mice (Fig. 6).

### Discussion

Results demonstrate that the Cdt venom injection into the subcutaneous tissue of mice did not induce a significant inflammatory response, confirming earlier clinical and experimental observations.[1,3,11] The mild oedematous response induced by the venom was not dose-dependent, transient and comparable to the oedematogenic response induced by serotonin or histamine into the hind paw of rats.[12] The oedema induced by Cdt venom was completely abolished by pretreatment with an antihistamine drug. These findings are corroborated by the observation of Vargaftig *et al.*[13] that antihistamine drugs inhibit the increased vascular permeability induced by this venom. Taken together, these results suggest that histamine may be the main mediator of the acute phase of the mild inflammatory response induced by Cdt venom.

The main toxin found in Cdt venom is crotoxin which is composed of two subunits, crotapotin and phospholipase $A_2$.[14] Phospholipase $A_2$ ($PLA_2$) present in several snakes venoms is endowed with potent oedema forming activity.[15–17] However, the mild oedematous response of crude Cdt venom might be the result of a low enzymatic activity of $PLA_2$ when it is associated with crotapotin.[18] Furthermore, it has been demonstrated

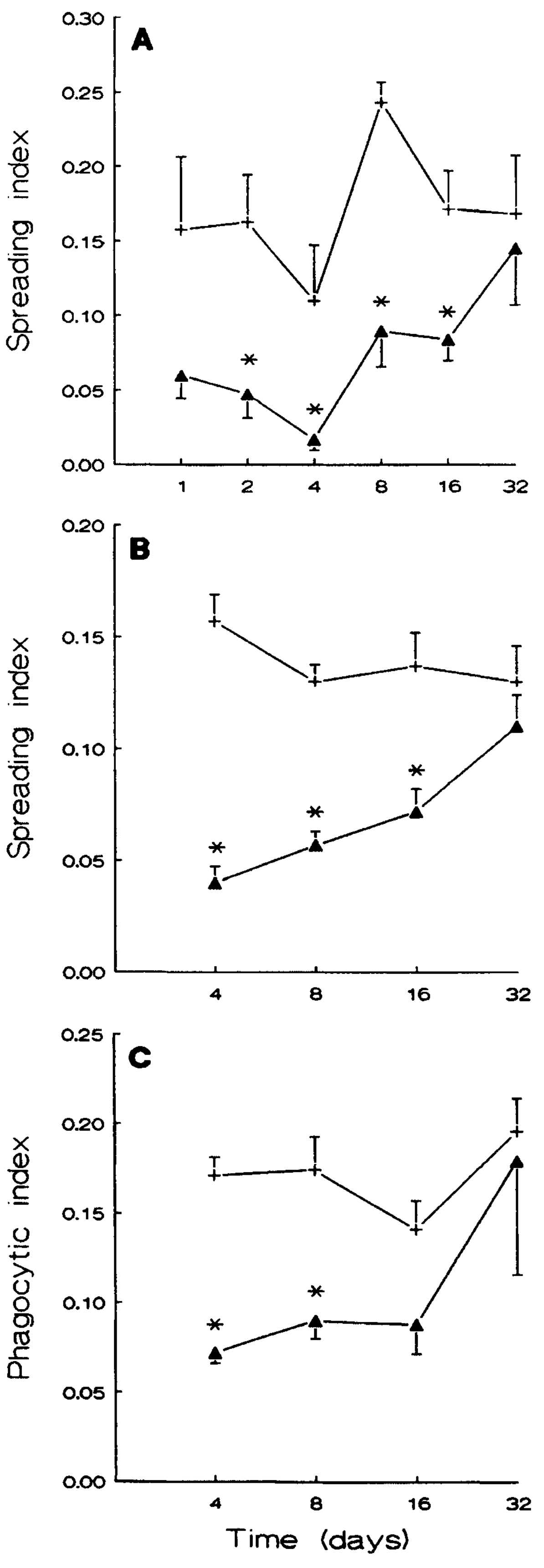

FIG. 5. Effect of the injection of *C.d.terrificus* venom i.p. (A) or s.c. (B,C) on the spreading (A,B) and phagocytic (C) activities of resident peritoneal macrophages of mice. Each point is the mean ± S.E.M. of six to eight mice. *$p < 0.05$. +, Control; ▲, experiment.

**Table 2.** Total and differential counts of cells of the peritoneal exudate obtained 4 h after the i.p. injection of thioglycolate, from mice pre-injected 1 h earlier with *C.d.terrificus* (Cdt) venom (1.5 μg, s.c.), or with saline (control)

| Groups | Cell count ($\times 10^6$/ml) | | |
|---|---|---|---|
| | Total | Differential | |
| | | Mono | PMN |
| Saline | 3.77 ± 0.52 | 1.04 ± 0.18 | 2.73 ± 0.39 |
| Cdt | 2.55 ± 0.82* | 1.15 ± 0.37 | 1.40 ± 0.44* |

Mono = mononuclear cells; PMN = polymorphonuclear cells.
Each point is the mean ± S.E.M. of five mice. *$p < 0.05$.

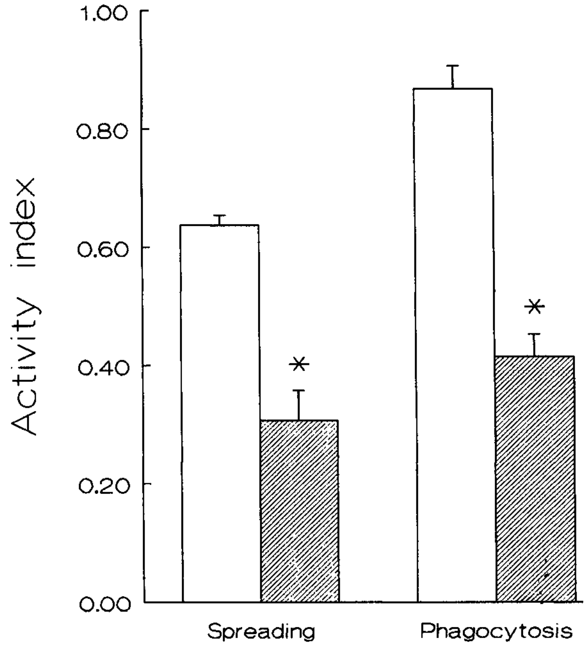

FIG. 6. Spreading and phagocytic activity index of murine peritoneal macrophages elicited by thioglycolate (i.p.) and simultaneous injection of *C.d.terrificus* venom (1.5 μg, s.c.) or saline (control). Each point is the mean ± S.E.M. of five mice. *$p < 0.05$. □, Control; ▨, experiment.

that crotapotin has a marked inhibitory effect on the carrageenin-induced paw oedema, a multi-mediated inflammatory response.[5]

The investigation of the Cdt venom on some of the macrophage functions was suggested by the observation that this venom has inhibitory effects in some inflammatory models[4,5] and by the fact that macrophages play a pivotal role in these phenomena.[19,20]

The Cdt venom showed an inhibitory effect on the spreading and the phagocytic activities of peritoneal macrophages. At concentrations used in this experiment, this venom did not induce alterations in cell viability *in vitro*, as opposed to the effect of some cytotoxins from other snake species which, by affecting the plasmatic membrane permeability, may cause cell lysis.[21] These observations suggest a down-regulatory property of the venom on the investigated functions of macrophages, which was also shown *in vivo*.

Although, in general, snake venoms are rapidly removed from circulation,[22,23] the inhibitory effects of Cdt venom observed on macrophage functions lasted for several days. The low turnover of peritoneal macrophages in mice (around 15 days)[24] could explain the long-lasting down-regulatory effect of the venom on these cells. This low turnover of these cells might also account for the progressive reduction of the inhibitory effect induced by the venom.

On the other hand, the inhibitory effect of Cdt venom on macrophage functions might be a consequence of the generation by the venom of serum factors, since the envenomation by *Crotalus durissus terrificus* can cause renal failure,[1] and the serum from rats with renal failure inhibits, *in vitro*, the phagocytic activity of peritoneal macrophages.[25] However, sera obtained from mice injected with Cdt venom did not alter, *in vitro*, the spreading ability of macrophages. The inhibitory effect of the venom on the spreading index of peritoneal macrophage from athymic Nu/Nu mice suggests that this phenomenon is not mediated by lymphocyte-released products.

The fact that, when added *in vitro*, Cdt venom did not inhibit the spreading and phagocytic capacities of macrophages elicited with thioglycollate but it was able to inhibit these activities in macrophages obtained from animals simultaneously injected with Cdt venom (s.c.) and thioglycollate (i.p.) suggests that the venom interferes with the activation of macrophages but not with the activity of those already elicited.

In fact, Cdt venom also inhibited the acute inflammatory response induced by thioglycollate, demonstrated by the reduction of inflammatory cell migration to the peritoneal cavity of animals injected with this drug plus venom. Preliminary data also indicate that Cdt venom significantly reduces the peritoneal cell migration induced by zymozan (Sousa e Silva and Cury, unpublished observations).

As inflammatory leukocyte migration is mediated by cytokines, and this includes those liberated by macrophages[26–30] there is reason to speculate that Cdt venom affects the inflammatory process by inhibiting the liberation of cytokines. This hypothesis is under investigation.

### References

1. Rosenfeld G. Symptomatology, pathology and treatment of snake bites in South America. In: Bücherl W, Buckley E, eds. *Venomous Animals and Their Venoms, vol. 2.* New York: Academic Press, 1971; 345–384.
2. Efrati P. Symptomatology, pathology and treatment of the bites of Viperid snakes. In: Lee CY, ed. *Handbook of Experimental Pharmacology, vol. 52, Snake Venoms.* Berlin: Springer-Verlag, 1979; 956–977.
3. Brazil V. Serumtherapia anti-ophidica. *Rev Méd S Paulo*; **15**: 197–227.
4. Giorgi R, Bernardi MM, Cury Y. Analgesic effect evoked by low molecular weight substances extracted from *Crotalus durrissus terrificus* venom. *Toxicon* 1993; **31**: 1257–1266.
5. Landucci ECT, Antunes E, Donato JL, *et al.* Inhibition of carrageenin-induced rat paw oedema by crotapotin, a polypeptide complexed with phospholipase $A_2$. *Br J Pharmacol* 1995; **114**: 578–583.
6. Winder CV, Wax I, Been MA. Rapid foot volume measurements of unanesthetized rats and the question of the phenylbutazone effect on anaphylactoid edema. *Arch Int Pharmacodyn* 1957; **112**: 335–340.
7. Rosenfeld G. Método rápido de coloração de esfregaços de sangue. Noções práticas sobre corantes pancrômicos e estudos de diversos fatores. *Mem Inst Butantan* 1947; **20**: 315–328.
8. Rabinovitch M, DeStefano MJ. Macrophage spreading *in vitro*. *Exp Cell Res* 1973; **77**: 323–334.
9. Edelson PJ, Cohn ZA. Effects of concanavalin A on mouse peritoneal macrophages. 1. Stimulation of endocytic activity and inhibition of phago-lysosome formation. *J Exp Med* 1974; **140**: 1364–1403.
10. Berquó ES, de Souza JMP, Gotlieb SLD. Bioestatística, São Paulo: E.P.U., 1981.
11. Amorim MF, Franco de Mello R, Saliba F. Envenenamento botrópico e crotálico. *Mem Inst Butantan* 1951; **23**: 63–108.
12. Bonta IL, Vargaftig BB, Bohn GM. Snake venoms as an experimental tool to induce and study models of microvessel damage. In: Lee CY, ed. *Handbook of Experimental Pharmacology, vol. 52, Snake Venoms.* Berlin: Springer-Verlag, 1979; 956–977.
13. Vargaftig BB, Bhargava N, Bonta IL. Haemorrhagic and permeability increasing effects of *Bothrops jararaca* and other Crotalidae venoms as related to amine or kinin release. *Agents and Actions* 1974; **413**: 163–168.
14. Habermann E, Breithaupt H. The crotoxin complex—an example of biochemical and pharmacological protein complementation. *Toxicon* 1978; **16**: 19–30.
15. Wang JP, Teng CM. Rat paw oedema and mast cell degranulation caused by two phospholipase $A_2$ enzymes isolated from *Trimeresurus mucrosquamatus* venom. *J Pharm Pharmacol* 1990; **42**: 846–850.
16. Tan NH, Saifuddin MN, Young WY. The edema inducing activity of phospholipase $A_2$ enzymes. *Biochemistry International* 1991; **23**: 175–181.
17. Llorett S, Moreno JG. Oedema formation and degranulation of mast cells by phospholipase $A_2$ purified from porcine pancreas and snake venoms. *Toxicon* 1993; **31**: 949–956.
18. Breithaupt H. Enzymatic characteristics of *Crotalus* phospholipase $A_2$ and crotoxin complex. *Toxicon* 1978; **14**: 221–223.
19. Ferreira SH. Are macrophages the body's alarm cells? *Agents and Actions* 1980; **10**: 229–230.
20. Francischi JN, Lorenzetti BB, Ferreira SH. Interleukin-I mimics the hyperalgesia induced by a factor obtained by macrophage lysis. *Brazilian J Med Biol Res* 1988; **21**: 321–331.
21. Karlsson E. Chemistry of protein toxins in snake venoms. In: Lee CY, ed. *Handbook of Experimental Pharmacology, vol. 52, Snake venoms.* Berlin: Springer-Verlag, 1979; 480–546.
22. Theakston RDG, Lloyd-Jones MJ, Reid HA. Micro-ELISA for detecting and assaying snake venom and venom-antibody. *Lancet* 1977; **ii**: 639–641.
23. Domingos MO, Takehara HA, Laing G, *et al.* Detection and neutralization of *B. jararaca* venom in mice. *Brazilian J Med Biol Res* 1994; **27**: 2613–2622.
24. van Furth R. Origin and turnover of monocytes and macrophages. *Curr Top Pathol* 1989; **79**: 125–150.
25. Ori M, Seguro AC, Rocha AS. Efeito inibidor do soro de ratos com insuficiência renal aguda e crônica sobre a atividade fagocitária *in vitro*. *Rev Inst Med Trop São Paulo* 1990; **32**: 409–413.
26. Peveri P, Walz A, De Wald B, Baggliolini M. A novel neutrophil-activating factor produced by human mononuclear phagocytes. *J Exp Med* 1988; **167**: 1547–1559.
27. Merril WW, Naegel GP, Matthag RA, Reynolds HY. Alveolar macrophage-derived chemotactic factor. Kinetics of *in vitro* production and partial characterization. *J Clin Invest* 1980; **65**: 269–276.
28. Russo M. The role of macrophages in the chemotactic response of polymorphonuclear leukocytes to bacterial lipopolysacharides. *Proc Soc Exp Biol Med* 1980; **164**: 326–330.
29. Souza GEP, Cunha FQ, Mello R, Ferreira SH. Neutrophil migration induced by inflammatory stimuli is reduced by macrophage depletion. *Agents and Actions* 1988; **24**: 377–380.
30. Dias Baruffi M, Cunha F, Ferreira SH, Roque-Barreira MC. Macrophage-released neutrophil chemotactic factor [MNCF] induces PMN-neutrophil migration through lectin-like activity. *Agents and Actions* 1993; **38** [Special Issue]: C54–C56.

ACKNOWLEDGEMENTS. The authors thank Dr Eva M. A. Kelen for critically reading the manuscript and Ms Neusa T. Picon for technical support.

**Received 11 October 1995;**
**accepted in revised form 15 November 1995**
